# Geographic Distribution of *Leishmania* Species in Ecuador Based on the Cytochrome *B* Gene Sequence Analysis

**DOI:** 10.1371/journal.pntd.0004844

**Published:** 2016-07-13

**Authors:** Hirotomo Kato, Eduardo A. Gomez, Luiggi Martini-Robles, Jenny Muzzio, Lenin Velez, Manuel Calvopiña, Daniel Romero-Alvarez, Tatsuyuki Mimori, Hiroshi Uezato, Yoshihisa Hashiguchi

**Affiliations:** 1 Division of Medical Zoology, Department of Infection and Immunity, Jichi Medical University, Tochigi, Japan; 2 Laboratory of Parasitology, Department of Disease Control, Graduate School of Veterinary Medicine, Hokkaido University, Hokkaido, Japan; 3 Departamento de Parasitologia y Medicina Tropical, Facultad de Ciencias Medicas, Universidad Catolica de Santiago de Guayaquil, Guayaquil, Ecuador; 4 Departamento de Parasitologia, Insitituto de Investigacion de Salud Publica, Guayaquil, Ecuador; 5 Centro de Biomedicina, Facultad de Medicina, Universidad Central del Ecuador, Quito, Ecuador; 6 Department of Microbiology, Faculty of Life Sciences, Graduate School of Health Sciences, Kumamoto University, Kumamoto, Japan; 7 Department of Dermatology, Faculty of Medicine, University of the Ryukyus, Okinawa, Japan; Universidade Federal de Minas Gerais, BRAZIL

## Abstract

A countrywide epidemiological study was performed to elucidate the current geographic distribution of causative species of cutaneous leishmaniasis (CL) in Ecuador by using FTA card-spotted samples and smear slides as DNA sources. Putative *Leishmania* in 165 samples collected from patients with CL in 16 provinces of Ecuador were examined at the species level based on the cytochrome *b* gene sequence analysis. Of these, 125 samples were successfully identified as *Leishmania (Viannia) guyanensis*, *L*. *(V*.*) braziliensis*, *L*. *(V*.*) naiffi*, *L*. *(V*.*) lainsoni*, and *L*. *(Leishmania) mexicana*. Two dominant species, *L*. *(V*.*) guyanensis* and *L*. *(V*.*) braziliensis*, were widely distributed in Pacific coast subtropical and Amazonian tropical areas, respectively. Recently reported *L*. *(V*.*) naiffi* and *L*. *(V*.*) lainsoni* were identified in Amazonian areas, and *L*. *(L*.*) mexicana* was identified in an Andean highland area. Importantly, the present study demonstrated that cases of *L*. *(V*.*) braziliensis* infection are increasing in Pacific coast areas.

## Author Summary

In Ecuador, leishmaniasis is a major public health concern reported in 21 of 24 provinces of the country, and eight *Leishmania* species, *Leishmania* (*Leishmania*) *mexicana*, *L*. *(L*.*) amazonensis*, *L*. *(L*.*) major*-like, *L*. *(Viannia) guyanensis*, *L*. *(V*.*) panamensis*, *L*. *(V*.*) braziliensis*, *L*. *(V*.*) naiffi*, and *L*. *(V*.*) lainsoni*, have been identified as causative agents of human cutaneous (CL) and mucocutaneous leishmaniases (MCL). Causative parasite species for CL have been identified as *L*. *(V*.*) guyanensis*, *L*. *(V*.*) panamensis*, *L*. *(V*.*) braziliensis*, and *L*. *(L*.*) amazonensis* in Pacific coast areas, *L*. *(L*.*) mexicana* and *L*. *(L*.*) major*-like in Andean highland areas, and *L*. *(V*.*) guyanensis*, *L*. *(V*.*) braziliensis*, *L*. *(V*.*) naiffi*, and *L*. *(V*.*) lainsoni* in Amazonian areas. In the present study, a countrywide epidemiological survey was performed to elucidate the current geographic distribution of causative species of CL in Ecuador, by using FTA card-spotted samples and smear slides as DNA sources. Putative *Leishmania* in 165 samples collected from patients with CL in 16 provinces of Ecuador were examined based on the cytochrome *b* gene sequence analysis. From these, 125 samples were identified, of which two dominant species, *L*. *(V*.*) guyanensis* and *L*. *(V*.*) braziliensis*, were widely distributed in Pacific coast subtropical and Amazonian tropical areas, respectively. Importantly, the present study demonstrated that cases of *L*. *(V*.*) braziliensis* infection are increasing in Pacific coast areas.

## Introduction

Leishmaniasis is caused by protozoan parasites of the genus *Leishmania*, which is further divided into two subgenera, *Leishmania* (*Leishmania*) and *Leishmania* (*Viannia*). The disease is widely distributed around the world, especially in tropical and subtropical areas, affecting at least 12 million people in 98 countries [1]. Approximately 20 *Leishmania* species are known to be pathogenic to humans, and the infecting species is the major determinant of clinical outcome [1]. Therefore, identification of the parasite species in endemic areas is important for both appropriate treatment and prognosis.

In Ecuador, leishmaniasis is a major public health concern reported in 21 of 24 provinces of the country, in Pacific coast subtropical areas, Amazonian tropical areas and Andean highland areas [2–4]. During 2010 and 2014, 6,608 cases were registered in the Ministry of Public Health, Ecuador, ranging yearly from 899 to 1,629 (average 1,321.6), and in 2014, 262 (22.1%) of the 1,183 cases were derived from Pichincha province, followed by Santo Domingo de los Tsáchilas (148 cases, 12.5%), Esmeraldas (136 cases, 11.5%), Orellana (94 cases, 7.9%), Sucumbios (88 cases, 7.4%), and Morona Santiago (87 cases, 7.4%) provinces (Departamento de Epidemiologia, Ministerio de Salud Publica, 2014). Currently, eight *Leishmania* species, *Leishmania* (*Leishmania*) *mexicana*, *L*. *(L*.*) amazonensis*, *L*. *(L*.*) major*-like, *L*. *(Viannia) guyanensis*, *L*. *(V*.*) panamensis*, *L*. *(V*.*) braziliensis*, *L*. *(V*.*) naiffi*, and *L*. *(V*.*) lainsoni*, have been identified as causative agents of human cutaneous (CL) and mucocutaneous leishmaniases (MCL) in Ecuador [3, 5–9]. In Pacific coast areas, causative parasite species for CL have been identified as *L*. *(V*.*) guyanensis*, *L*. *(V*.*) panamensis*, *L*. *(V*.*) braziliensis*, and *L*. *(L*.*) amazonensis* [3, 5–9]. In Andean highland areas, *L*. *(L*.*) mexicana* and *L*. *(L*.*) major*-like have been reported as causative species for Andean-type CL, of which *L*. *(L*.*) mexicana* is dominant [3–7]. In Amazonian areas, *L*. *(V*.*) guyanensis*, *L*. *(V*.*) braziliensis*, *L*. *(V*.*) naiffi*, and more recently, *L*. *(V*.*) lainsoni* have been identified as causative agents for CL and MCL [5–9].

Currently, molecular biological methods are widely used for identification of *Leishmania* species using DNA extracted from clinical samples of patients’ lesions, and they have become a powerful tool for epidemiological studies of leishmaniasis [10–12]. DNA extracted from Giemsa-stained smears obtained from patients’ skin lesions, which have been used for the microscopic diagnosis to detect parasites in the lesions, has also been used as a template for detection and identification of *Leishmania*, although the sensitivity is not so high because of limitations of the DNA source [13–17]. Recently, to facilitate sample collection and DNA extraction processes, an FTA card, a filter paper that readily lyses spotted materials and fixes nucleic acids, was used for direct sampling of patients’ samples in an epidemiological study of leishmaniasis, and its usability for field epidemiology was reported [18–20]. In the present study, a countrywide epidemiological survey was performed to elucidate the current geographic distribution of causative species of CL in Ecuador, by using FTA card-spotted samples and smear slides as DNA sources.

## Materials and Methods

### Sample collection

During 2010 and 2015, clinical samples were collected from patients suspected of having CL at 41 sites in 16 provinces of Ecuador: Province of Esmeraldas: 1. Mataje, 2. Pampanal de Bolívar, 3. San Lorenzo, 4. Esmeraldas, 5. Atacames, and 6. Sabalito; Province of Manabi: 7. Pedernales, 8. San Isidro, 9. Junin, 10. Jipijapa, and 11. Montalvo; Province of Santa Elena: 12. Manglaralto; Province of Imbabura: 13. Cielo Verde; Province of Pichincha: 14. Puerto Quito, 15. Pedro Vicente Maldonado, 16. Los Bancos, 17. Nanegalito, 18. Pachijal, and 19. Quinche; Province of Santo Domingo: 20. Valle Hermoso, and 21. Chiguilpe; Province of Bolivar: 22. Balsapamba,; Province of Los Rios: 23. Quevedo; Province of Chimborazo: 24. Huigra; Province of Cañar: 25. La Troncal; Province of Guayas: 26. El Triunfo, 27. Naranjal, and 28. Balao; Province of El Oro: 29. Santa Rosa; Province of Scumbios: 30. Cascales, 31. Lago Agrio, 32. Putumayo, and 33. Palma Roja; Province of Orellana: 34. Coca, 35. Shangrila, 36. La Joya de los Sachas, and 37. Dayuma; Province of Pastaza: 38. Puyo, and 39. Arajuno; Province of Zamora-Chinchipe: 40. Palanda, and 41. Zumba (S1 Fig). Tissue samples were taken by scraping the margins of active lesions of a patient, spotted onto an FTA Classic Card (Whatman, Newton Center, MA) and stored at room temperature. Two-mm-diameter disks were punched out from each filter paper and washed three times with an FTA Purification Reagent (Whatman) and once with Tris-EDTA buffer. The disks were air-dried and directly subjected to PCR amplification. For the extraction of DNA from Giemsa-stained smears obtained from skin lesions (ulcers and/or nodules) on CL patients, 30 μl of DNA extraction buffer [150 mM NaCl, 10 mM Tris-HCl (pH 8.0), 10 mM EDTA and 0.1% sodium dodecyl sulfate (SDS)] containing 100 μg/ml of proteinase K were spotted on each smear and mixed well, and detached tissue materials in the DNA extraction buffer were transferred to 1.5 ml tubes. The sample was incubated at 37°C overnight, and heated at 95°C for 5 min. Each 0.5-μl portion was directly used as a template for PCR.

### Identification of *Leishmania* species

*Leishmania* species were identified by cytochrome *b* (*cyt* b) gene sequence analysis [18, 19]. PCR amplification with a pair of specific primers, L.cyt-AS (5'-GCGGAGAGRARGAAAAGGC-3') and L.cyt-AR (5'-CCACTCATAAATATACTATA-3'), was performed with 30 cycles of denaturation (95°C, 1 min), annealing (55°C, 1 min) and polymerization (72°C, 1 min) using Ampdirect Plus reagent (Shimadzu Biotech, Tsukuba, Japan). Each 0.5-μl portion of the PCR product was reamplified with L.cyt-S (5'-GGTGTAGGTTTTAGTYTAGG-3') and L.cyt-R (5'-CTACAATAAACAAATCATAATATRCAATT-3'). The products were cloned into the pGEM-T Easy Vector System (Promega, Madison, WI) and sequences were determined by the dideoxy chain termination method using a BigDye Terminator v3.1 Cycle Sequencing Kit (Applied Biosystems, Foster City, CA). The parasite species were identified based on the homology with *cyt* b gene sequences from *Leishmania* reference strains. The result was confirmed by a phylogenetic analysis and a distant matrix using the program MEGA (Molecular Evolutionary Genetics Analysis) version 5.2.

### Phylogenetic analysis

The *Leishmania cyt* b gene sequences were aligned with CLUSTAL W software [21] and examined using the program MEGA version 5.2 [22]. Phylogenetic trees were constructed by the maximum likelihood (ML) method with Kimura 2 parameter [22]. Branch support for ML tree was calculated using the bootstrapping method with 1,000 replicates in MEGA 5.2 [22]. The database for phylogenetic analyses consisted of *cyt* b gene sequences from 12 *Leishmania* species, *L*. *(L*.*) donovani* (GenBank accession number: AB095957), *L*. *(L*.*) infantum* (AB095958), *L*. *(L*.*) tropica* (AB095960), *L*. *(L*.*) major* (AB095961), *L*. *(L*.*) mexicana* (AB095963, EF579906), *L*. *(L*.*) amazonensis* (AB095964), *L*. *(V*.*) braziliensis* (AB095966, AB434681, AB434682, AB095967), *L*. *(V*.*) panamensis* (AB095968), *L*. *(V*.*) guyanensis* (AB095969, EF579905), *L*. *(V*.*) naiffi* (AB433279), *L*. *(V*.*) lainsoni* (AB433280) and *L*. *(V*.*) shawi* (AB433281).

### Ethics statement

Sample collection was performed by local physicians and well-trained laboratory technicians with the approval of the research ethics committee of the Graduate School of Veterinary Medicine, Hokkaido University (license number: vet26-4). Informed consent was obtained from the adult subjects and from the children’s parents or guardians, prior to collection of diagnostic materials at each health center of the Ecuadorian Ministry of Health. Signed consent was obtained after explanation of the process of diagnosis and *Leishmania* species analysis during routine diagnosis carried out at rural health centers, following the guidelines of the Ethics Committee of the Ministry of Health, Ecuador. The subjects studied were volunteers in routine diagnosis/screening and treatment programs promoted by the Ministry. All routine laboratory examinations were carried out free of charge, and treatment with specific drug (Glucantime) was also offered free of charge at each health center of the Ministry.

## Results

**Fig 1:**
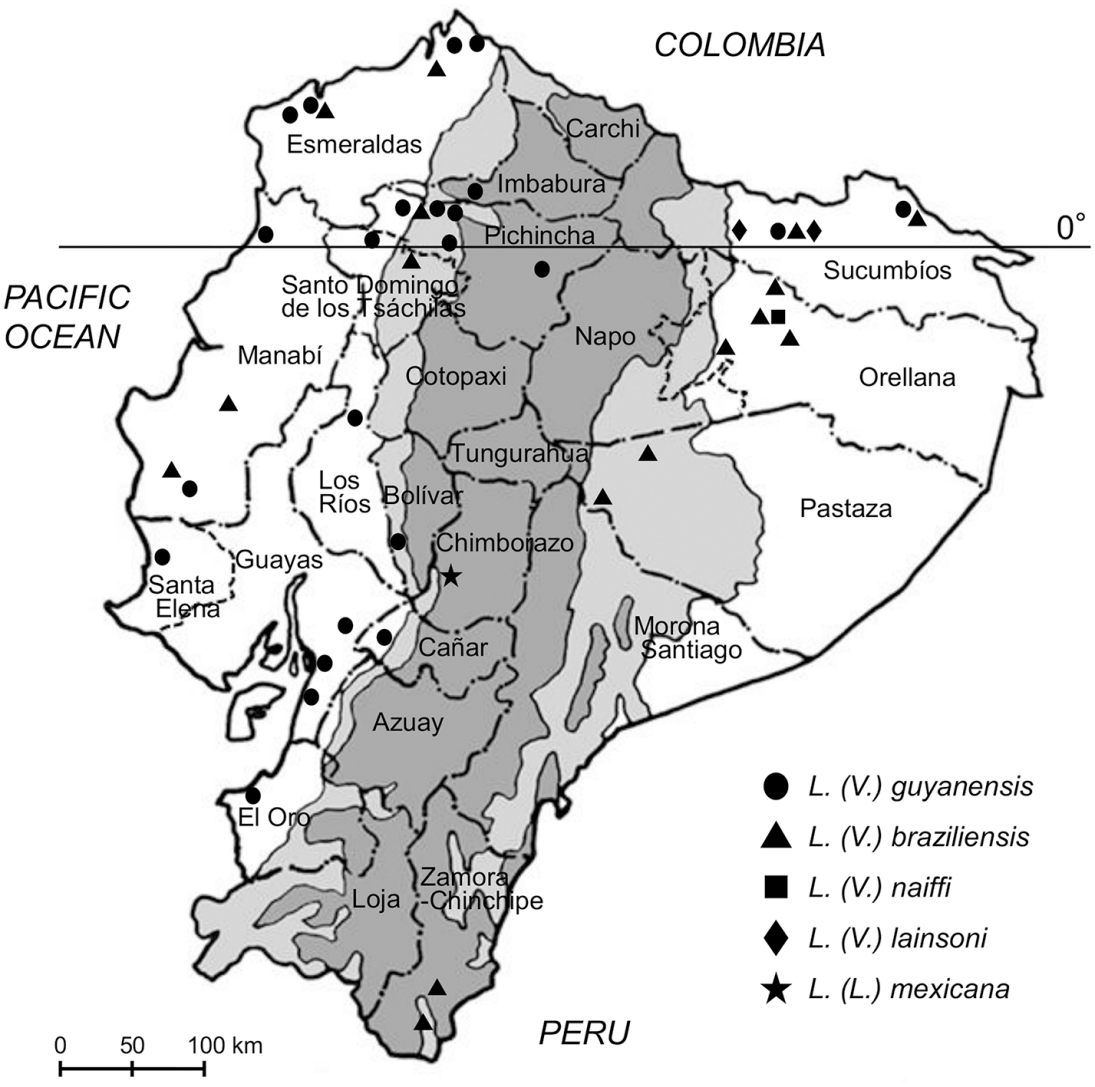
Geographic distribution of *Leishmania (Viannia) guyanensis*, *L*. *(V*.*) braziliensis*, *L*. *(V*.*) naiffi*, *L*. *(V*.*) lainsoni*, and *L*. *(Leishmania) mexicana* in Ecuador. The dark gray areas show the Andean plateau (>1,000 m altitude), and the light gray areas show highland jungle or Andean slopes (400–1,000 m elevation).

A total of 165 samples (162 FTA cards and three smear samples) were collected from patients living in endemic areas of Ecuador who were suspected of having cutaneous leishmaniasis. The samples were subjected to PCR targeting the leishmanial *cyt* b gene, and the amplification was repeated, not more than twice, to obtain gene fragments of the parasites from samples negative in the first PCR. Leishmanial *cyt* b gene fragments were obtained from 125 (123 FTA cards and two smear samples) of 165 patients’ samples (75.8%). Parasites were identified to species on the basis of *cyt* b gene sequence analysis (S2 Fig) [18, 19]. The nucleotide sequence data are available in the DDBJ/EMBL/GenBank databases under the accession numbers LC055618-LC055621 and LC153160-LC153277. The distribution of *Leishmania* species by province in Ecuador is presented in Table 1 and Fig 1, and by ecological region (Pacific coast subtropical, Andean highland, and Amazonian tropical areas) in Table 2. Among the 125 samples, two dominant species, *L*. *(V*.*) guyanensis* (74.4%) and *L*. *(V*.*) braziliensis* (20.0%), were widely distributed in the Pacific coast subtropical and Amazonian tropical areas, respectively (Table 1).

**Table 1:** Distribution of analyzed samples of *Leishmania* species by province in Ecuador.

Province	Locality	*Leishmania* species*					Total
		Lg	Lb	Ln	Ll	Lm	
Bolivar	Balsapamba	1	0	0	0	0	1
Cañar	La Troncal	3	0	0	0	0	3
Chimborazo	Huigra	0	0	0	0	1	1
El Oro	Santa Rosa	2	0	0	0	0	2
Esmeraldas	Atacames	1	0	0	0	0	1
	Esmeraldas	3	1	0	0	0	4
	Mataje	1	0	0	0	0	1
	Pampanal de Bolívar	1	0	0	0	0	1
	San Lorenzo	0	1	0	0	0	1
Guayas	Balao	1	0	0	0	0	1
	El Triunfo	1	0	0	0	0	1
	Naranjal	4	0	0	0	0	4
Imbabura	Cielo Verde	1	0	0	0	0	1
Los Rios	Quevedo	1	0	0	0	0	1
Manabi	Jipijapa	0	1	0	0	0	1
	Junin	1	1	0	0	0	2
	Montalvo	1	0	0	0	0	1
	Pedernales	1	0	0	0	0	1
Orellana	Coca	0	1	0	0	0	1
	Dayuma	0	2	0	0	0	2
	La Joya de los Sachas	0	1	0	0	0	1
	Shangrila	0	0	4		0	4
Pastaza	Arajuno	0	1	0	0	0	1
	Puyo	0	1	0	0	0	1
Pichincha	Los Bancos	8	1	0	0	0	9
	Nanegalito	3	0	0	0	0	3
	Pachijal	1	0	0	0	0	1
	Pedro Vicente Maldonado	38	5	0	0	0	43
	Puerto Quito	3	1	0	0	0	4
	Quinche	1	0	0	0	0	1
Santa Elena	Manglaralto	2	0	0	0	0	2
Santo Domingo de los Tsáchilas	Chiguilpe	0	1	0	0	0	1
	Valle Hermoso	10	0	0	0	0	10
Sucumbíos	Cascales	0	0	0	2	0	2
	Lago Agrio	3	3	0	0	0	6
	Palma Roja	0	1	0	0	0	1
	Putumayo	1	0	0	0	0	1
Zamora-Chinchipe	Palanda	0	2	0	0	0	2
	Zumba	0	1	0	0	0	1
Total		93	25	4	2	1	125

*Lg, *L*. *(V*.*)* guyanensis; Lb, *L*. *(V*.*) braziliensis*; Ln, *L*. *(V*.*) naiffi*; Ll, *L*. *(V*.*) lainsoni*; Lm, *L*. *(L*.*) mexicana*.

**Table 2:** Distribution of analyzed samples of *Leishmania* species by ecological region in Ecuador.

Species	Pacific coast	Andes	Amazonia	Total
*L*. *(V*.*) guyanensis*	89	0	4	93
*L*. *(V*.*) braziliensis*	12	0	13	25
*L*. *(V*.*) naiffi*	0	0	4	4
*L*. *(V*.*) lainsoni*	0	0	2	2
*L*. *(L*.*) mexicana*	0	1	0	1
Total	101	1	23	125

In Pacific coast subtropical provinces (Bolivar, Cañar, El Oro, Esmeraldas, Guayas, Imbabura, Los Rios, Manabi, Pichincha, Santa Elena, and Santo Domingo de los Tsáchilas), 61 of 101 samples (60.4%) were collected from Pichincha province. Although four *Leishmania* species, *L*. *(V*.*) guyanensis*, *L*. *(V*.*) panamensis*, *L*. *(V*.*) braziliensis*, and *L*. *(L*.*) amazonensis* have been reported as causative agents in subtropical areas in Ecuador, all the *Leishmania* samples from such areas in this study were identified as *L*. *(V*.*) guyanensis* and *L*. *(V*.*) braziliensis*, of which *L*. *(V*.*) guyanensis* was dominant (Table 2, Fig 1). *Leishmania (V*.*) braziliensis* was identified in northern and central provinces (Esmeraldas, Manabi, Pichincha, and Santo Domingo de los Tsáchilas) (Table 1, Fig 1). In Andean highland areas, a CL case from the province of Chimborazo was examined and infecting parasites identified as *L*. *(L*.*) mexicana*. In Amazonian tropical provinces (Orellana, Pastaza, Sucumbíos, Zamora-Chinchipe), *L*. *(V*.*) braziliensis* was identified as the dominant species as reported previously [5, 8, 9]. In addition, other species, *L*. *(V*.*) guyanensis*, *L*. *(V*.*) naiffi*, and *L*. *(V*.*) lainsoni* were identified in this study; all were reported previously in Ecuador, the latter two rather recently [7, 8] (Table 1, Fig 1). Cases of *L*. *(V*.*) naiffi* infection were identified in the previously reported area (Shangrila) in Orellana province, and *L*. *(V*.*) lainsoni* infections were identified in Sucumbíos province. In the southern Amazonian province (Zamora-Chinchipe), only *L*. *(V*.*) braziliensis* was identified.

In the present study, all patients had typical ulcerative and/or nodular cutaneous lesions; none had mucosal or mucocutaneous lesions. The number of cutaneous lesions per patient ranged from one to six, and the diameter of lesions ranged from 0.5 to 5cm. The one *Leishmania (L*.*) mexicana* infection in an Andean area caused a typical small ulcerative lesion (0.5cm), the so-called “Andean-type CL” [4]. No marked characteristic differences in cutaneous lesions among causative *Leishmania* species were observed.

## Discussion

A countrywide survey was conducted to elucidate the current geographic distribution of causative species of CL in Ecuador on the basis of *cyt* b gene analysis. Using minimally invasive sampling methods such as FTA card collections and smear slides, causative agents were successfully identified in 125 patients from 41 areas of 16 provinces in Ecuador. The results indicate that *L*. *(V*.*) guyanensis* and *L*. *(V*.*) braziliensis* are widely distributed in Pacific coast subtropical and Amazonian tropical areas, respectively. The data obtained also suggest that CL cases caused by *L*. *(V*.*) braziliensis* are increasing in Pacific coast areas. Distributions of *L*. *(V*.*) naiffi* and *L*. *(V*.*) lainsoni*, both of which have been identified recently in the Ecuadorian Amazon, were confirmed, and *L*. *(L*.*) mexicana* was identified in an Andean area.

Although five *Leishmania* species were identified in this study, previous studies reported distribution of three other species, *L*. *(V*.*) panamensis* and *L*. *(L*.*) amazonensis* in Pacific coast subtropical areas, and *L*. *(L*.*) major*-like in Andean highland areas in Ecuador [3–5]. Of these, wide distribution of *L*. *(V*.*) panamensis* was identified by multilocus enzyme electrophoresis (MLEE) in Pacific coast areas [3, 5], whereas *L*. *(L*.*) amazonensis*, for which samples were not examined in this study, was identified from only a few areas [3, 5]. *Leishmania (V*.*) panamensis* is very closely-related to *L*. *(V*.*) guyanensis*, and a previous study questioned the distinctness of the two species by MLEE and genetic analyses of *Leishmania* isolates in Ecuador [23]. Previous studies reported that *L*. *(V*.*) panamensis* and *L*. *(V*.*) guyanensis* were separated in distinct clades by phylogenetic analysis targeting the *cyt* b gene [24, 25]; however, multiple genetic analyses of Ecuadorian isolates identified as *L*. *(V*.*) panamensis* or *L*. *(V*.*) guyanensis* by MLEE revealed discordant results among targeted genes, which is in agreement with a previous enzymatic and genetic analyses of the two species [23]. Therefore, it is speculated that *L*. *(V*.*) guyanensis* identified in this study includes *Leishmania* species previously identified as *L*. *(V*.*) guyanensis* and *L*. *(V*.*) panamensis* by MLEE. Since the present classification of *Leishmania* species has been defined by MLEE and genetic analyses of *Leishmania* suggested that the number of species could be very large, reclassification of *Leishmania* species including *L*. *(V*.*) panamensis* and *L*. *(V*.*) guyanensis* may be needed using extensive multiple genetic analyses [26, 27]. *Leishmania (L*.*) major*-like has been reported in Andean areas as a minor species causing CL [4]; however, this infection has not been detected recently. The present study examined only one sample from an Andean area, and the infection parasite was identified as *L*. *(L*.*) mexicana*, the major causative species in Andean highland areas [3–5]. The characteristic *cyt* b gene sequence, which composes a separate clade from other *L*. *(L*.*) mexicana* strains including reference strains by a phylogenetic analysis [18], was confirmed. In Amazonian areas, four species, *L*. *(V*.*) braziliensis*, *L*. *(V*.*) guyanensis*, *L*. *(V*.*) naiffi*, and *L*. *(V*.*) lainsoni*, were identified, of which distributions of the latter two species have been reported recently [7, 8]. Distribution of *L*. *(V*.*) lainsoni*, the most recently reported species in Ecuador [7, 8], was recorded in several areas in the Sucumbíos and Orellana provinces. On the other hand, cases of *L*. *(V*.*) naiffi* were identified only in a military training camp at Shangrila, Orellana province, as reported previously [7]. The vector species of *L*. *(V*.*) naiffi* has been identified as *Lutzomyia (Lu*.*) tortura* in the same area [7]. Although natural infection of *Lu*. *tortura* by *L*. *(V*.*) naiffi* has also been detected in Arajuno, Pastaza province [6], no human cases of infection with *L*. *(V*.*) naiffi* have been reported in this area. It may be interesting to compare its transmission cycle, including reservoir animals and the vector’s host preferences in the two areas to understand different occurrences of CL caused by *L*. *(V*.*) naiffi*.

The present study revealed wide distribution of *L*. *(V*.*) guyanensis* and *L*. *(V*.*) braziliensis* in Pacific coast and Amazonian areas. Wide distribution of the two species has been reported in other South American countries [28], reflecting the broad vector and reservoir ranges of these *Leishmania* species. One of the most important findings of this study is that cases of *L*. *(V*.*) braziliensis* infection seem to be increasing in Pacific coast areas of Ecuador when compared to past studies [3, 5]. Distribution of *L*. *(V*.*) braziliensis* and its sand fly vectors may be expanding in these areas. Alternatively, parasite isolation, which is required for MLEE, may be inefficient in *L*. *(V*.*) braziliensis* when compared to other species, resulting in fewer identifications of this species in past studies. The procedures for isolation of *L*. *(V*.*) braziliensis* parasites from patient’s lesions and its maintenance/culture *in vitro* are very difficult compared to other *Leishmania* species because of the extremely limited presence of amastigotes in the lesions and/or maladaptation of the species to an artificial culture medium. Genetic analysis of directly sampled materials as conducted in this study can overcome this issue. Since infection by *L*. *(V*.*) braziliensis* is associated with destructive mucocutaneous lesions [1], continuous surveillance will be needed. At present, MCL has been reported rarely in the Pacific coast areas in Ecuador. Several factors such as patients’ genetic background and/or pathogenicity of parasite strains may be associated with the formation of mucocutaneous lesions.

The present countrywide surveillance revealed the current geographic distribution of causative species of CL in Ecuador. The less-invasive and easy-to-use FTA card will be a useful tool for further continuous monitoring of prevalent *Leishmania* species. Together with prevalent parasite species, vector and reservoir research will be needed since this information is limited in Ecuador despite its importance for control of leishmaniasis.

## Supporting Information

S1 Fig Sample collection sites in Ecuador. The dark gray areas show the Andean plateau (>1,000 m altitude), and the light gray areas show highland jungle or Andean slopes (400–1,000 m elevation). 1. Mataje, 2. Pampanal de Bolívar, 3. San Lorenzo, 4. Esmeraldas, 5. Atacames, and 6. Sabalito, Province of Esmeraldas; 7. Pedernales, 8. San Isidro, 9. Junin, 10. Jipijapa, and 11. Montalvo, Province of Manabi; 12. Manglaralto, Province of Santa Elena; 13. Cielo Verde, Province of Imbabura; 14. Puerto Quito, 15. Pedro Vicente Maldonado, 16. Los Bancos, 17. Nanegalito, 18. Pachijal, and 19. Quinche, Province of Pichincha; 20. Valle Hermoso, and 21. Chiguilpe, Province of Santo Domingo; 22. Balsapamba, Province of Bolivar; 23. Quevedo, Province of Los Rios; 24. Huigra, Province of Chimborazo; 25. La Troncal, Province of Cañar; 26. El Triunfo, 27. Naranjal, and 28. Balao, Province of Guayas; 29. Santa Rosa, Province of El Oro; 30. Cascales, 31. Lago Agrio, 32. Putumayo, and 33. Palma Roja, Province of Scumbios; 34. Coca, 35. Shangrila, 36. La Joya de los Sachas, and 37. Dayuma, Province of Orellana; 38. Puyo, and 39. Arajuno, Province of Pastaza; 40. Palanda, and 41. Zumba, Province of Zamora-Chinchipe.
(TIF)

S2 Fig Phylogenetic tree of *cyt* b gene sequences among species. Leishmanial *cyt* b genes were amplified and sequenced from patients with cutaneous leishmaniasis, and a phylogenetic analysis of *cyt* b gene sequences was performed by the neighbor-joining method together with sequences from 12 *Leishmania* species. The scale bar represents 0.01% divergence. Bootstrap values are shown above or below branches.
(TIF)

S1 Checklist STROBE Statement. (DOC)

## References

[1] Alvar J, Vélez ID, Bern C, Herrero M, Desjeux P, Cano J, Jannin J, den Boer M. WHO Leishmaniasis Control Team., Leishmaniasis worldwide and global estimates of its incidence. PLoS One. 2012; 7:e35671 10.1371/journal.pone.0035671 22693548 PMC3365071

[2] Gomez EA, Hashiguchi Y. Monthly variation in natural infection of the sand-fly *Lutzomyia ayacuchensis* with *Leishmania mexicana* in an endemic focus in the Ecuadorian Andes. Ann Trop Med Parasitol. 1991; 85:407–411. 1796881 10.1080/00034983.1991.11812584

[3] Hashiguchi Y, Gómez Landires EA. A review of leishmaniasis in Ecuador. Bull Pan Am Health Organ. 1991; 25:64–76. 2054554

[4] Hashiguchi Y, Gomez EA, de Coronel VV, Mimori T, Kawabata M, Furuya M, Nonaka S, Takaoka H, Alexander JB, Quizhpe AM, Grimaldi G Jr, Kreutzer RD, Tesh RB. Andean leishmaniasis in Ecuador caused by infection with *Leishmania mexicana* and *L*. *major*-like parasites. Am J Trop Med Hyg. 1991; 44:205–217. 1672799 10.4269/ajtmh.1991.44.205

[5] Calvopiña M, Armijos RX, Hashiguchi Y. Epidemiology of leishmaniasis in Ecuador: current status of knowledge—a review. Mem Inst Oswaldo Cruz. 2004; 99:663–672. 15654419 10.1590/s0074-02762004000700001

[6] Kato H, Gomez EA, Yamamoto Y, Calvopiña M, Guevara AG, Marco JD, Barroso PA, Iwata H, Hashiguchi Y. Natural infection of *Lutzomyia tortura* with *Leishmania (Viannia) naiffi* in an Amazonian area of Ecuador. Am J Trop Med Hyg. 2008; 79:438–440. 18784239

[7] Kato H, Calvopiña M, Criollo H, Hashiguchi Y. First human cases of *Leishmania (Viannia) naiffi* infection in Ecuador and identification of its suspected vector species. Acta Trop. 2013; 128:710–713. 10.1016/j.actatropica.2013.09.001 24044975

[8] Kato H, Bone AE, Mimori T, Hashiguchi K, Shiguango GF, Gonzales SV, Velez LN, Guevara AG, Gomez EA, Hashiguchi Y. First human cases of *Leishmania (Viannia) lainsoni* infection and a search for the vector sand flies in Ecuador. PLoS Negl Trop Dis. In press. 10.1371/journal.pntd.0004728 PMC4871579 27191391

[9] Olalla HR, Velez LN, Kato H, Hashiguchi K, Caceres AG, Gomez EA, Zambrano FC, Romero-Álvarez DA, Guevara AG, Hashiguchi Y. An analysis of reported cases of leishmaniasis in the southern Ecuadorian Amazon region, 1986–2012. Acta Trop. 2015; 146:119–126. 10.1016/j.actatropica.2015.03.015 25796313

[10] Dujardin JC, Victoir K, De Doncker S, Guerbouj S, Arévalo J, Le Ray D. Molecular epidemiology and diagnosis of *Leishmania*: what have we learnt from genome structure, dynamics and function? Trans R Soc Trop Med Hyg. 2002; 96:S81–86. 12055856 10.1016/s0035-9203(02)90056-8

[11] Vega-López F. Diagnosis of cutaneous leishmaniasis. Curr Opin Infect Dis. 2003; 16:97–101. 12734442 10.1097/00001432-200304000-00006

[12] Reithinger R, Dujardin JC. Molecular diagnosis of leishmaniasis: current status and future applications. J Clin Microbiol. 2007; 45:21–25. 17093038 10.1128/JCM.02029-06 PMC1828971

[13] Motazedian H, Karamian M, Noyes HA, Ardehali S. DNA extraction and amplification of *Leishmania* from archived, Giemsa-stained slides, for the diagnosis of cutaneous leishmaniasis by PCR. Ann Trop Med Parasitol. 2002; 96:31–34. 11989531 10.1179/000349802125000484

[14] Al-Jawabreh A, Schoenian G, Hamarsheh O, Presber W. Clinical diagnosis of cutaneous leishmaniasis: a comparison study between standardized graded direct microscopy and ITS1-PCR of Giemsa-stained smears. Acta Trop. 2006; 99:55–61. 16920056 10.1016/j.actatropica.2006.07.001

[15] Brustoloni YM, Lima RB, da Cunha RV, Dorval ME, Oshiro ET, de Oliveira AL, Pirmez C. Sensitivity and specificity of polymerase chain reaction in Giemsa-stained slides for diagnosis of visceral leishmaniasis in children. Mem Inst Oswaldo Cruz. 2007; 102:497–500. 17612771 10.1590/s0074-02762007005000036

[16] Khademvatan S, Neisi N, Maraghi S, Saki J. Diagnosis and identification of *Leishmania* spp. from Giemsa-stained slides, by real-time PCR and melting curve analysis in south-west of Iran. Ann Trop Med Parasitol. 2011; 105:559–565. 10.1179/2047773211Y.0000000014 22325815 PMC4089809

[17] Koarashi Y, Cáceres AG, Zúniga Saca FM, Palacios Flores EE, Celis Trujillo A, Abanto Alvares JL, Yoshimatsu K, Arikawa J, Katakura K, Hashiguchi Y, Kato H. Identification of causative *Leishmania* species in Giemsa-stained smears prepared from patients with cutaneous leishmaniasis in Peru using PCR-RFLP. Acta Trop. 2016; 158:83–87. 10.1016/j.actatropica.2016.02.024 26943992

[18] Kato H, Cáceres AG, Mimori T, Ishimaru Y, Sayed AS, Fujita M, Iwata H, Uezato H, Velez LN, Gomez EA, Hashiguchi Y. Use of FTA cards for direct sampling of patients' lesions in the ecological study of cutaneous leishmaniasis. J Clin Microbiol. 2010; 48:3661–3665. 10.1128/JCM.00498-10 20720027 PMC2953078

[19] Kato H, Watanabe J, Mendoza Nieto I, Korenaga M, Hashiguchi Y. *Leishmania* species identification using FTA card sampling directly from patients' cutaneous lesions in the state of Lara, Venezuela. Trans R Soc Trop Med Hyg. 2011; 105:561–567. 10.1016/j.trstmh.2011.05.009 21907375

[20] Nzelu CO, Cáceres AG, Guerrero-Quincho S, Tineo-Villafuerte E, Rodriquez-Delfin L, Mimori T, Uezato H, Katakura K, Gomez EA, Guevara AG, Hashiguchi Y, Kato H. A rapid molecular diagnosis of cutaneous leishmaniasis by colorimetric malachite green-loop-mediated isothermal amplification (LAMP) combined with an FTA card as a direct sampling tool. Acta Trop. 2016; 153:116–119. 10.1016/j.actatropica.2015.10.013 26516109

[21] Thompson JD, Higgins DG, Gibson TJ. CLUSTAL W: improving the sensitivity of progressive multiple sequence alignment through sequence weighting, position-specific gap penalties and weight matrix choice. Nucleic Acids Res. 1994; 22:4673–4680. 7984417 10.1093/nar/22.22.4673 PMC308517

[22] Tamura K, Peterson D, Peterson N, Stecher G, Nei M, Kumar S. MEGA5: molecular evolutionary genetics analysis using maximum likelihood, evolutionary distance, and maximum parsimony methods. Mol Biol Evol. 2011; 28:2731–2739. 10.1093/molbev/msr121 21546353 PMC3203626

[23] Bañuls AL, Jonquieres R, Guerrini F, Le Pont F, Barrera C, Espinel I, Guderian R, Echeverria R, Tibayrenc M. Genetic analysis of *Leishmania* parasites in Ecuador: are *Leishmania (Viannia) panamensis* and *Leishmania (V*.*) guyanensis* distinct taxa? Am J Trop Med Hyg. 1999; 61:838–845. 10586922 10.4269/ajtmh.1999.61.838

[24] Luyo-Acero GE, Uezato H, Oshiro M, Takei K, Kariya K, Katakura K, Gomez-Landires E, Hashiguchi Y, Nonaka S. Sequence variation of the cytochrome *b* gene of various human infecting members of the genus *Leishmania* and their phylogeny. Parasitology. 2004; 128:483–491. 15180316 10.1017/s0031182004004792

[25] Asato Y, Oshiro M, Myint CK, Yamamoto Y, Kato H, Marco JD, Mimori T, Gomez EA, Hashiguchi Y, Uezato H. Phylogenic analysis of the genus *Leishmania* by cytochrome *b* gene sequencing. Exp Parasitol. 2009; 121:352–361. 10.1016/j.exppara.2008.12.013 19159626

[26] Fraga J, Montalvo AM, De Doncker S, Dujardin JC, Van der Auwera G. Phylogeny of *Leishmania* species based on the heat-shock protein 70 gene. Infect Genet Evol. 2010; 10:238–245. 10.1016/j.meegid.2009.11.007 19913110

[27] Schönian G, Mauricio I, Cupolillo E. Is it time to revise the nomenclature of *Leishmania*? Trends Parasitol. 2010; 26:466–469. 10.1016/j.pt.2010.06.013 20609626

[28] Grimaldi G Jr, David JR, McMahon-Pratt D. Identification and distribution of New World *Leishmania* species characterized by serodeme analysis using monoclonal antibodies. Am J Trop Med Hyg. 1987; 36:270–287. 3826486 10.4269/ajtmh.1987.36.270

