# Supplementary figures and images for: Geographic Distribution of Leishmania Species in Ecuador Based on the Cytochrome B Gene Sequence Analysis
Source: PLoS Negl Trop Dis. 2016 Jul 13;10(7):e0004844. doi: 10.1371/journal.pntd.0004844 (PMC4943627; doi:10.1371/journal.pntd.0004844)

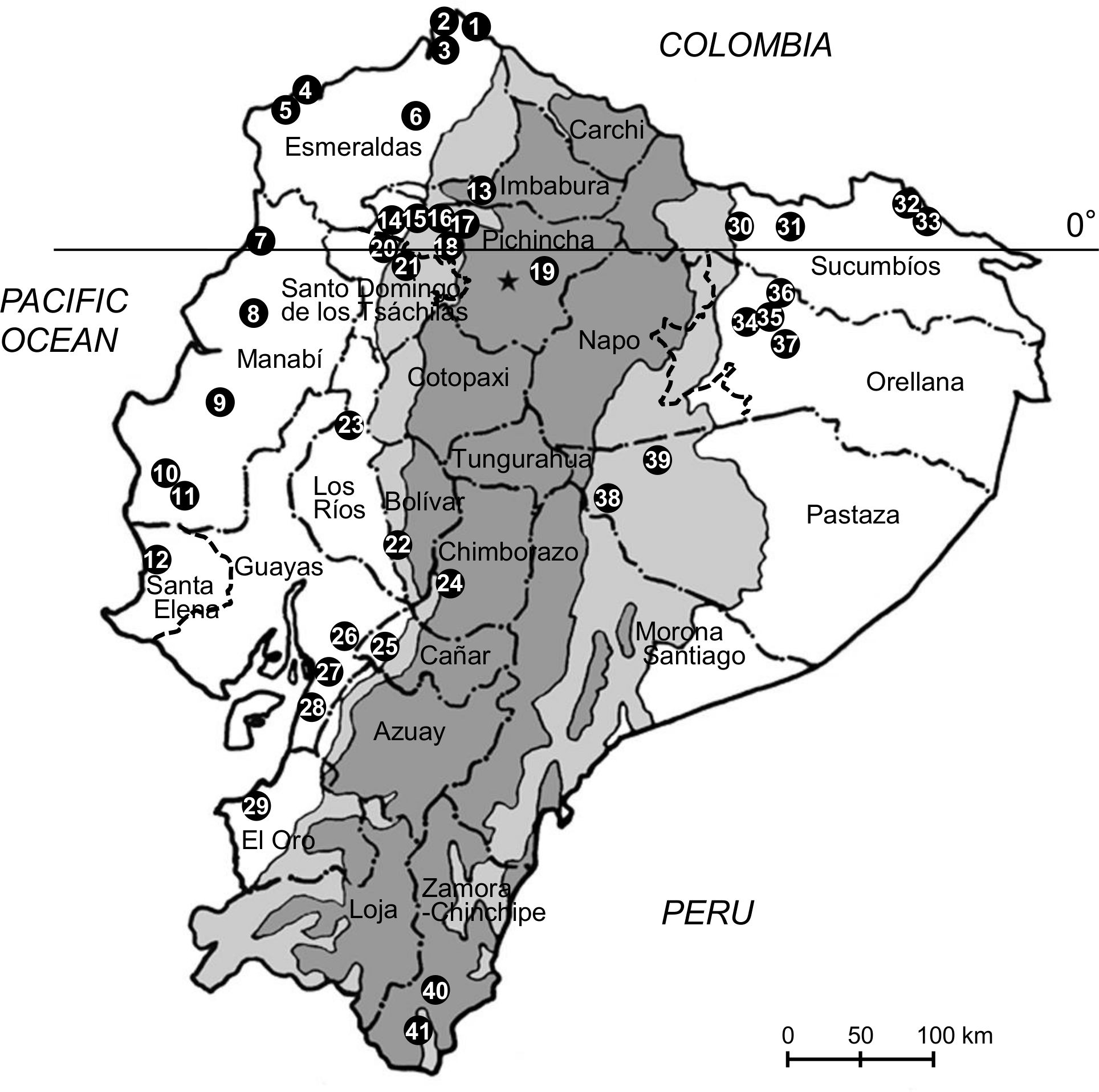

Supplement: S1 Fig — The dark gray areas show the Andean plateau (>1,000 m altitude), and the light gray areas show highland jungle or Andean slopes (400–1,000 m elevation). 1. Mataje, 2. Pampanal de Bolívar, 3. San Lorenzo, 4. Esmeraldas, 5. Atacames, and 6. Sabalito, Province of Esmeraldas; 7. Pedernales, 8. San Isidro, 9. Junin, 10. Jipijapa, and 11. Montalvo, Province of Manabi; 12. Manglaralto, Province of Santa Elena; 13. Cielo Verde, Province of Imbabura; 14. Puerto Quito, 15. Pedro Vicente Maldonado, 16. Los Bancos, 17. Nanegalito, 18. Pachijal, and 19. Quinche, Province of Pichincha; 20. Valle Hermoso, and 21. Chiguilpe, Province of Santo Domingo; 22. Balsapamba, Province of Bolivar; 23. Quevedo, Province of Los Rios; 24. Huigra, Province of Chimborazo; 25. La Troncal, Province of Cañar; 26. El Triunfo, 27. Naranjal, and 28. Balao, Province of Guayas; 29. Santa Rosa, Province of El Oro; 30. Cascales, 31. Lago Agrio, 32. Putumayo, and 33. Palma Roja, Province of Scumbios; 34. Coca, 35. Shangrila, 36. La Joya de los Sachas, and 37. Dayuma, Province of Orellana; 38. Puyo, and 39. Arajuno, Province of Pastaza; 40. Palanda, and 41. Zumba, Province of Zamora-Chinchipe. (TIF) [file pntd.0004844.s001.tif]

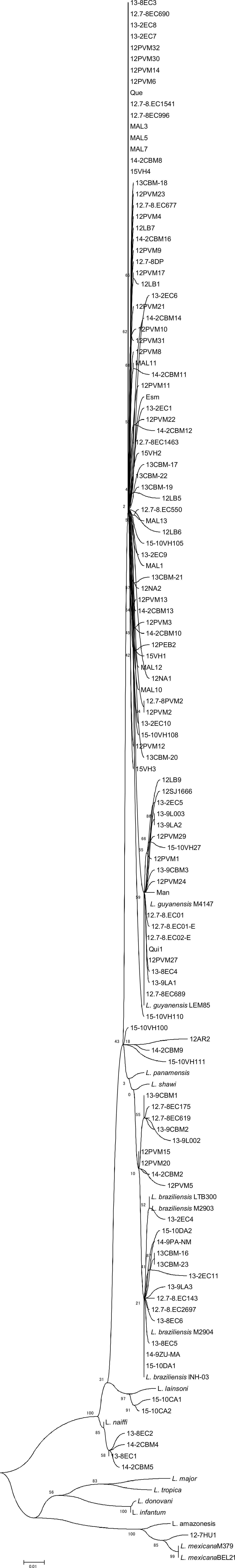

Supplement: S2 Fig — Leishmanial cyt b genes were amplified and sequenced from patients with cutaneous leishmaniasis, and a phylogenetic analysis of cyt b gene sequences was performed by the neighbor-joining method together with sequences from 12 Leishmania species. The scale bar represents 0.01% divergence. Bootstrap values are shown above or below branches. (TIF) [file pntd.0004844.s002.tif]
